# Direct and indirect associations between mental health and motivational indicators with physical activity among Lithuanian adolescents

**DOI:** 10.3389/fpsyg.2025.1492548

**Published:** 2025-04-07

**Authors:** Brigita Mieziene, Tomas Venckunas, Arunas Emeljanovas, Laima Trinkuniene, Kristina Zaicenkoviene, Daiva Vizbaraite

**Affiliations:** ^1^Department of Physical and Social Education, Lithuanian Sports University, Kaunas, Lithuania; ^2^Institute of Sport Sciences and Innovations, Lithuanian Sports University, Kaunas, Lithuania; ^3^Department of Coaching, Lithuanian Sports University, Kaunas, Lithuania; ^4^Department of Health and Rehabilitation, Lithuanian Sports University, Kaunas, Lithuania

**Keywords:** adolescents, moderate-to-vigorous physical activity, social support, psychological well-being, psychological distress

## Abstract

**Background:**

Considering the low engagement of contemporary adolescents in physical activity (PA), apparently, PA still has a low priority for adolescents, who are the only ones making decisions and performing behavior. So, analysis of more proximal factors that lay on the personal and interpersonal levels as well as psychological mechanisms forming PA behavior is important.

**Methods:**

The population-based cross-sectional study included 4,924 5th to 12th-grade school students. Among them, 50.9% were girls. The mean age of study participants varied from 11 to 19 years [mean 14.08 (2.21)]. Moderate-to-vigorous physical activity was measured by four items out of the IPAQ-SF questionnaire. Psychological well-being was assessed using The World Health Organization Five Well-Being Index (WHO-5) 5-item questionnaire. Psychological distress has been assessed by Kessler’s six-item scale. Social support in terms of family and friends social support has been assessed by a 13-item subscale of Sallis’ Support for Exercise Survey. Body mass index (BMI) was calculated by dividing body mass (kg) by height-squared (m^2^).

**Results:**

Higher motivation for MVPA was predicted by higher family (*β* = 0.653) but not friends‘support and both mental health indicators – higher psychological well-being (β = 0.049) and lower psychological distress (*β* = −0.078) were linked to higher motivation for physical activity, regardless the covariates. Higher motivation (*β* = 0.137), greater psychological well-being (β = 0.580) with the greatest magnitude, and lower psychological distress (β = −0.293) contributed to the greater MVPA.

**Conclusion:**

Family but not friends’ support for physical activity, greater psychological well-being, and lower psychological distress have direct and indirect effects on greater moderate-to-vigorous physical activity in adolescents.

## Introduction

Moderate to vigorous physical activity (MVPA) has proved to be an informative indicator of current and/or future physical health, specifically of physical fitness and body composition (Drenowatz et al., 2016), mental health (McMahon et al., 2017; Pastor et al., 2021), cognitive performance and academic achievements (Esteban-Cornejo et al., 2015), and chronic diseases (Anderson and Durstine, 2019; Pastor et al., 2021). Moreover, engagement in physical activity (PA) is a way to socialize with peers, as well as establish other social connections (Jonsson et al., 2017; Li and Zizzi, 2018), which are important factors affecting mental and physical health *per se*, particularly in children and adolescents (Román et al., 2023). Also, speaking the language of economy, it is worth noting that physical inactivity causes a significant financial burden for societies (Ding et al., 2016).

The World Health Organization (WHO) recommends at least an hour of MVPA daily for children and adolescents to stay healthy and develop normally (Bull and Willumsen, 2020). However, extra physical activities, including resistance exercises, are recommended to improve muscle strength, manage weight, and render additional health benefits (Bull et al., 2020). Meanwhile, children and adolescents around the world struggle to achieve at least a minimal level of these guidelines. While numbers of (in)sufficient PA among adolescents differ between sources [e.g., WHO 2019 data reports that 38% of 11–15-year-old Lithuanians meet the recommendation – “National Recommendations on Physical Activity for Health (2021)”]; while another epidemiological study at the same time revealed much lower (16%) number of sufficiently active 11-18-year-old Lithuanians (Mieziene et al., 2021), lack of adolescents’ PA is among the leading public health concern nationally and globally. To this end, a recent Lancet study presented that globally, as much as 80% of adolescents fail to meet the WHO PA recommendations (Sluijs et al., 2021).

PA is one of the key factors of psychological well-being - an indicator of mental health. Studies showed that regularly exercising students have the highest psychological well-being in terms of sense of happiness, positive emotions, good relationships, lower isolation, and stronger social support than those who do not exercise (Kovalenko et al., 2020). On the other hand, contemporary adolescents around the world have a lot of burdens compromising their mental health, e.g., COVID-19-associated restrictions, which increased social isolation (Okuyama et al., 2021), societal tensions due to pending or active military conflicts, or socialization problems related to screen (electronics) abuse. Recent studies report increasing rates of mental health disorders among adolescents (Beaudry et al., 2021). Therefore, the emerging picture unveils that current global changes negatively affect both PA and mental health and additionally suggests that low PA and poor mental health of adolescents are not just interrelated, but could well be that they amplify one another in a kind of vicious circle. In other words, while mental health state might be and is usually studied as an outcome of changes in PA, it also might be that compromised mental health causes or leads to insufficient PA in modern adolescents.

Enhancement of PA on a global level is important. Countries’ governments have already made efforts such as building PA-friendly infrastructure and promoting PE programs, which may contribute to increase PA. However, PA is still of low priority among adolescents who, because of their specific age period of gradually gaining independence from parents/guardians and establishing themselves yet stronger among peers, basically are the ones deciding on how much they are finally active in fact. On the other hand, as adolescents are anyway a part of and their decisions and actions are not totally independent from their social environment, consequently social support could be one of the key drivers for their PA behavior (Sluijs et al., 2021). So, analysis of more proximal factors that lay on the personal and interpersonal levels and psychological mechanisms forming PA behavior is important to understand the possible leverage of introducing healthy behaviors such as sufficient PA among adolescents nowadays. Besides emotional, financial, informational, or instrumental support that the social environment may offer, it also helps form PA-friendly attitudes turning into motivation for PA (Ajzen and Schmidt, 2020), which becomes a part of adolescent personality and drives their behavior into introgression of sufficient levels of PA into daily schedules.

### The present study

The hypothesized links we proposed in this study are based on the following considerations. Motivation or willingness to engage in behavior is considered the most proximal predictor of intentional and conscious behavior in accordance with the different health-behavior models like Self-Determination Theory (SDT; Ryan and Deci, 2000), Theory of Planned Behavior (Ajzen and Schmidt, 2020), or Social Cognitive Theory (Bandura, 1999). It might be driven by cognitive (like perceived benefits) and affective (like pleasure) motives (Bandura, 1999; Conner et al., 2013). So, in the present study, we considered personal motivation for PA to be the main direct predictor of volitional MVPA. All of the above mentioned theories of behavior change emphasize the importance of supporting significant others in behavioral motivation (Ryan and Deci, 2000). Motivation itself is formed in a social environment, with family and friends as social groups in adolescence are of utmost importance in developing motivation for PA (Ajzen and Schmidt, 2020; Morrissey et al., 2015). So, social support for PA from family and friends here are predictors of motivation to be physically active. Moreover, in accordance with the Social Cognitive theory, behavior indirectly may be also affected by the current emotional state besides other factors, and in fact, this relationship is bi-directional (Bandura, 1999). Given that not only PA impact mental health, but vice versa is probable as well, the present study seeks to explore the impact of current mental health on the willingness to be engaged in PA. Both phenomena address psychological states and are supposed to be interrelated. Although mental health indirectly, through motivation, is supposed to predict MVPA, testing for direct links might reveal if it is personal/internal motivation that mediates the effect of mental health or other mediators are of higher/larger importance.

**Figure 1:**
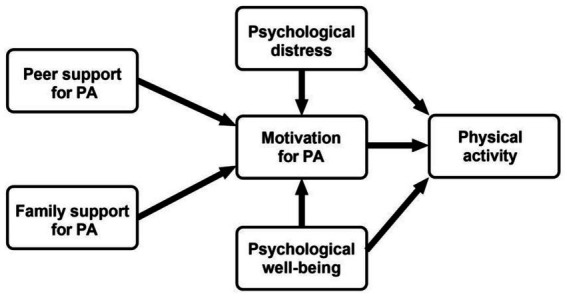
Proposed model illustrating the interplay of psychosocial factors, mental health, and physical activity.

Based on the theoretical background, we hypothesize that increased physical activity levels are directly associated with higher motivation for physical activity and better mental health. Furthermore, we propose that physical activity is either directly or indirectly through the mediating role of motivation, linked to improved mental health and enhanced social support (Figure 1).

The study aims to explore the direct and indirect relationship of social support, mental health, and motivation to be physically active with moderate-to-vigorous physical activity in a population-based sample of Lithuanian adolescents.

## Methods

### Participants and sampling

The population-based cross-sectional study included 4,924 school students of 5th to 12th grade. Among them, 50.9% were girls, and 49.1% were boys per their gender self-reports. The age of study participants was 11 to 19 [mean (SD) 14.08 (2.21)] years.

### Study design and procedure

Nested sampling was employed to select participants for the study. From all ten counties of Lithuania, at least one school from the county’s center and at least one school from the region were randomly selected. Next, the school administration was contacted to agree on study performance. If the school administration refused to participate in the study, a similar school in the county was selected. In total, 42 schools were included. From each school, one class per grade (from 5th to 12th) was selected and all students in selected classes filled out the online study questionnaires (for about 30 min to fill in) following the provided link unless their parents had not signed parental consent. Data was collected in 2022 from September to December. Permission to conduct the study was obtained from the Lithuanian Sports University’s Committee of Bioethics (permission no. 27720). The principles outlined in the Declaration of Helsinki were regarded in performing this study.

### Measurements

Moderate-to-vigorous physical activity was measured by four items out of the IPAQ-SF questionnaire (Craig et al., 2003). Items identified weekly frequency and duration per bout of moderate and vigorous physical activity.

Psychological well-being was assessed using The World Health Organization-Five Well-Being Index (WHO-5) 5-item questionnaire (Topp et al., 2015). The questionnaire identified the frequency of being active, vigorous, rested, relaxed, having interests, and being in good spirits. Answers ranged on the Likert scale from 0 – “none of the time” to 5 – “all of the time.” The summed total score was multiplied by 4 as proposed in the to have a range of scores from 0 to 100. The higher score indicated better psychological well-being. The WHO-5 is suitable for children aged 9 and above. The instrument has been found to have adequate validity in screening for depression and in measuring outcomes in clinical trials (Topp et al., 2015). The internal consistency in the current study was Cronbach *α* 0.872 indicating good internal validity. The Lithuanian version of WHO-5 has been used in several previous studies among adolescents (Jusienė et al., 2022; Slapšinskaitė et al., 2020).

Psychological distress has been assessed by Kessler’s six-item scale (Kessler et al., 2003). School students had to evaluate their nervousness, hopelessness, anxiety, restlessness or fidgety feelings, worthlessness, and depression on a scale from 0 (none of the time) to 4 (all the time). The total score was obtained by summing the scores for each item. A lower score indicates a lower level of psychological distress. The internal consistency of the scale was good (Cronbach α = 0.897). The instrument has been translated into Lithuanian and has been used in numerous previous studies among Lithuanian adolescents (Novak et al., 2016; Novak et al., 2017).

Social support in terms of family and friends social support has been assessed by a 13-item subscale of Sallis’ Support for Exercise Survey (Sallis et al., 1988). Identical items for two reference groups – family and friends – identified support for physical activity. Items addressed encouragement to be physically active (e.g., “Gave me rewards for exercising”), involvement in PA (e.g., “Offered to exercise with me”), and facilitation (e.g., “Changed their schedule so we could exercise together”) within the three recent months. Answers ranged from 1 – “Never” to 5 – “Very often”. Two negatively worded items (“Complained about the time I spend exercising” and “Criticized me or made fun of me for exercising”), were reversed before the calculation of the final score on each scale (Family Social Support and Friends Social Support). A higher score corresponded with higher perceived social support. The internal consistency for family social support was *α* = 0.87 and for friends’ social support was α = 0.89, indicating good internal consistency. The scale has not been previously used among Lithuanian populations. So, the double translation was performed and the final version of the questionnaire was evaluated by expert in the field.

Motivation for physical activity was assessed using a 9-item scale identifying school students’ attitudes toward exercising because of physical, and cognitive health motives, understanding the importance of PA, and satisfaction with PA (e.g., “Exercising improves health and well-being”). Each answer has four options: (1) “No”, (2) “Rather no than yes”, (3) “Rather yes than no”, and (4) “Yes”. The higher the score the more autonomous is the motivation to exercise. The internal consistency of this scale was Cronbach α = 0.916. The study authors constructed the scale based on the adjusted Pictorial Motivation Scale in Physical Activity (Reid et al., 2009). This study is the first one where the derived scale has been used.

Exploratory factor analysis with Promax rotation and principal Axis Factoring derived one factor (KMO = 0.927; df = 36; *p* < 0.001), explaining 57% of the variance. Confirmation factor analysis confirmed one-factor model (CFI = 0.971, TLI = 0.956, RMSEA = 0.082 [0.077–0.086], SRMR = 0.029; χ^2^ = 778.554, df = 24; *p* < 0.0001). The standardized estimates of item loads were from 0.688 to 0.807.

Body mass index (BMI) was calculated by dividing body mass (kg) by height-squared (m^2^), which were measured using digital weighing scales and stadiometers, respectively. Groups of BMI were allocated following Cole et al.’ (2000) reference norms for age and gender.

Gender (male/female) and age were identified by asking the students to click on the appropriate answer option or write down themselves.

### Statistical analysis

SPSS 28.0 (SPSS Inc., Chicago, IL, United States) and MPLUS 8.4 software were employed to analyze data. Descriptive statistics in terms of means (SD) and frequencies were used. Pearson correlation coefficient was employed to define interrelationships between study variables. Student t-test was used to determine the mean differences between the two groups. The chi-square test was employed to identify differences in frequency. Exploratory Factor Analysis (EFA) and Confirmatory Factor Analysis (CFA) were performed to identify the factor structure for the questionnaire.

For a hypothetical structural equation model for mediation, the following model fit indices were employed: (1) the root mean square error of approximation (RMSEA) and its 90% confidence interval (CI) - a population-based fit index that is insensitive to the sample size. Values of 0.01, 0.05, and 0.08 indicate excellent, good, and mediocre fit respectively; (2) the Standardized Root Mean Squared Residual (SRMR) - a direct assessment of how well an *a priori* model reproduces the sample data. Values <0.05 were considered to indicate a very good fit, and values <0.08 were interpreted as indicating a good fit; (3) the comparative fit index (CFI) and (4) Tucker–Lewis index (TLI) used to compare the fit of a hypothesized model with that of a baseline model (i.e., the model with the worst fit). Their values >0.90 indicate a good model fit, and values >0.95 indicate a very good model fit (Kline, 2023). Maximum likelihood (ML) estimation is used to estimate the parameters of an assumed probability distribution, given some observed data. Statistical significance was set at a *p*-value of less than 0.05.

## Results

Results of descriptive statistics (Table 1) revealed that among 11 to 19-year-old Lithuanians, 11.4% were overweight or obese. Overweight and obesity were more prevalent among boys than girls and reached almost 4% of the difference between genders. Two-thirds of adolescents had low psychological distress, as well as high psychological well-being. Meanwhile, more than one-third had high psychological distress, and ~ 20% had poor psychological well-being and as musch as ~10% had a risk of depression. These measures of mental health were worse in girls than boys. Among psychosocial indicators, perception of support for PA from family and friends, motivation for PA (which in total were above average) as well as PA itself were greater among boys than girls.

**Table 1:** Descriptive statistics of variables.

Study variables	Total	Girls	Boys	*p*
	% or mean (SD)			
BMI [mean (SD)]	20.59 (4.09)	20.29 (3.97)	20.90 (4.19)	<0.001
Not overweight	88.6	90.4	86.8	
Overweight	11.4	9.6	13.2	<0.001
PD [mean (SD)]	15.18 (5.16)	13.71 (5.12)	16.71 (4.72)	<0.001
High	36.0	47.6	23.9	
Low	64.0	52.4	76.1	<0.001
PWB [mean (SD)]	59.29 (22.04)	53.57 (22.04)	65.23 (20.41)	<0.001
Risk for depression	11.2	15.8	6.5	
Poor	22.5	29.1	15.6	
High	66.3	55.0	77.9	<0.001
Motivation [mean (SD)]	30.65 (5.77)	30.16 (5.75)	31.16 (5.75)	<0.001
Family support [mean (SD)]	2.52 (0.72)	2.47 (0.72)	2.57 (0.72)	<0.001
Friends support [mean (SD)]	2.65 (0.81)	2.56 (0.79)	2.74 (0.83)	<0.001
MVPA [METs; mean (SD)]	5,076 (3712)	4,310 (3439)	5,871 (3816)	<0.001

BMI, Body Mass Index; PD, Psychological Distress; PWB, Psychological Well-Being; MVPA, Moderate-to-Vigorous Physical Activity.

All studied variables (covariates not included) were interrelated (Table 2). The strongest relationships were found between the two indicators of mental health and the two indicators of support for PA. Higher psychological well-being is related to lower psychological distress and greater perception of family support is related to greater perception of peer support.

**Table 2:** Relationships between study variables (Pearson r).

	Motivation	Family support	Friends support	PWB	PD
Motivation	1				
Family’s support	0.306^**^	1			
Friends’ support	0.387^**^	0.477^**^	1		
PWB	0.256^**^	0.329^**^	0.268^**^	1	
PD	−0.247^**^	−0.222^**^	−0.177^**^	−0.501^**^	1
MVPA	0.308^**^	0.341^**^	0.374^**^	0.298^**^	−0.188^**^

PD, Psychological Distress; PWB, Psychological Well-Being; MVPA, Moderate-to-Vigorous Physical Activity. **p < 0.01.

Structural equation modeling was performed to reveal the direct and indirect relationships between mental health, psychosocial indicators, and MVPA (Table 3). Chi-square test of model fit retrieved value of χ^2^ = 1.108; df = 2; *p* = 0.574. RMSEA was equal to 0, with its 90% CI [0–0.024]. Both CFI and TLI values were equal to 1 and SRMR = 0.002. So, the model had a very good fit.

**Table 3:** Direct and Indirect relationships between moderate-to-vigorous physical activity and independent variables.

From	Through	To	Estimate of STDYX standartization	*p*
Direct relationships				
Family’s support		Motivation	0.653	<0.001
Friends’ support		Motivation	0.014	0.157
PWB		Motivation	0.049	<0.001
PD		Motivation	−0.078	<0.001
Covariates				
BMI (overweight and obese)		Motivation	−0.100	<0.001
Gender (boys)		Motivation	0.036	<0.001
Age		Motivation	0.016	0.100
R^2^ = 0.542; *p* < 0.001				
Motivation		MVPA	0.137	<0.001
PWB		MVPA	0.580	<0.001
PD		MVPA	−0.293	<0.001
Covariates				
BMI (overweight and obese)		MVPA	−0.034	0.008
Gender (boys)		MVPA	−030	0.018
Age		MVPA	−004	0.747
R^2^ = 0.288; *p* < 0.001				
Indirect relationships				
Family support	Motivation	MVPA	0.090	<0.001
Friends support	Motivation	MVPA	0.002	0.161
PWB	Motivation	MVPA	0.007	<0.001
PD	Motivation	MVPA	0.011	<0.001

BMI, Body Mass Index; PD, Psychological Distress; PWB, Psychological Well-Being; MVPA, Moderate-to-Vigorous Physical Activity.

Higher family but not friends’ support predicted higher motivation for MVPA, and both mental health indicators—namely higher psychological well-being and lower psychological distress—were linked to higher motivation for PA after adjusting for covariates. Family support had the greatest predictive power for motivation for PA. Among covariates, lower BMI and male gender predicted higher motivation but not age. In total, all included factors explained more than half of the variance in motivation for PA.

Mental health indices were also included among predictors of MVPA along with motivation for PA, and all three related/associated with MVPA. Higher motivation, greater psychological well-being with the greatest magnitude, and lower psychological distress contributed to the greater MVPA. Among covariates, being not overweight and male gender predicted greater MVPA. Among covariates, being not overweight and of male gender predicted greater MVPA. In total, motivation and mental health indicators along with sociodemographic and BMI explained almost 29% of variance in MVPA.

Analysis of the indirect relationships showed that motivation was a mediator between family support, psychological well-being, psychological distress, and MVPA, translating the indirect effects of mental health and psychosocial indicators into MVPA.

## Discussion

This cross-sectional study was aimed to explore direct and indirect correlates of the level of moderate-to-vigorous physical activity of adolescents. Among Lithuanian school students aged 11–19 years, higher MVPA was predicted by higher students’ motivation towards PA.

Motivation by health behavior change theories is considered one of the most proximal behavioral predictors (Ryan and Deci, 2000), followed by consciously formed intention leading to action (Ajzen, 2006). Empirical longitudinal and cross-sectional studies usually confirm this theoretical premise (Doré et al., 2020; Kalajas-Tilga et al., 2020; Zhang et al., 2021). Motivation itself contains attitudes towards behavior, and internal and external incentives (Ajzen and Schmidt, 2020). Formation of motivation is affected by social environment which for children and adolescents usually is their family and peers. So, motivation for PA in the current study translated the support provided by family, but not peers, to the actual level of PA. Both family and peers’ social support for physical activity were significantly related to higher MVPA in correlational analysis. However, in a complex analysis – structural equation modeling – peer‘s social support was not significantly related to MVPA either directly or indirectly. The underlying explanation might be that peers are a social group that tends to perform PA together. There are no educational incentives for interaction with peers. It is just the action. They do PA together because they have similar interests. Meanwhile, family members, especially senior ones, besides engaging in PA together, also usually educate youngsters on the importance of PA, and support their physical activities informationally, instrumentally, and financially. Another study illustrating this insight was performed among young cancer survivors, however in general the results could most likely be applied to other populations as well. The researchers found that parents perceive their role is to provide instrumental, informational, and emotional support (Felten-Barentsz et al., 2021). That is the way family support could translate into motivation for PA in adolescents and turn into their increased levels of PA.

Another issue might be that it takes time to accumulate the perception of social support, and as more prolonged interactions with peers usually start in life later compared to that with the members of the family, therefore a supportive effect of the latter could be captured in time earlier. The current study was cross-sectional, while in a longitudinal study, researchers found that support from both social groups was directly related to adolescents’ PA even after controlling predictors for each other (Morrissey et al., 2015). Similarly, the review of other studies on this topic indicated that both family and friends are important for involvement in PA. Moreover, encouragement for PA and engagement in PA together were the most frequent types of social support associated with PA in adolescents (Mendonça et al., 2014).

Another important PA-related factor is psychological well-being, which in the current study directly and indirectly predicted MVPA. The relationship between psychological well-being and PA is well-established in many other studies which are summarized in the recent meta-analysis (Buecker et al., 2021). The relationship between PA and well-being across the lifespan suggests that in adolescence, both PA and well-being tend to decline (Hyde et al., 2013). As rule, these analyzed studies consider PA as a predictor of psychological or other kind of well-being. However, the interaction between PA and well-being might be reciprocal. In the current study, it was presumed that the ongoing mental health state was primarily dictating behavior toward PA. In line with these findings, a prospective study confirms that mental well-being prognosticates PA: it was found that higher levels of mental well-being are associated with higher PA in one year (Román et al., 2023). The same presumption of direction in this study was applied to psychological distress. Similarly, an overview of overviews suggests that a higher level of PA is a predictor of lower symptoms of depression, anxiety, and distress across a wide range of adult populations (Singh et al., 2023). A study in adults with anxiety revealed that PA along with social support reduces psychological distress (Pereira-Payo et al., 2023). However, some longitudinal studies investigated the effect in the opposite direction. For instance, the LOOK study revealed that there is no evidence for a longitudinal effect of depression on PA. Meanwhile, cross-sectionally higher scores on depression and stress were related to lower PA (Olive et al., 2016). Further longitudinal and experimental/interventional studies are required to investigate the relationship between mental health and PA. However, it could be implied that a better mental state at a given time enables behaviors that might be less likely activated by being in worse mental health.

The current study also found that among covariates, being not overweight and male gender were related to higher motivation for PA and MVPA. Other studies are in line with these results showing that higher BMI is related to lower levels of PA (Sulemana et al., 2006). Moreover, decline in PA levels during adolescence in a longitudinal study was associated with an incline in BMI (Kimm et al., 2005). Observational studies show that PA interventions at school have the potential to reduce elevated BMI in school students (Jacob et al., 2021). Another study also illustrated the relationship between BMI and motivation for PA. Among motives for fitness, enjoyment, social, and competence, etc., only motives for improved appearance were significantly different between normal weight and overweight adolescents, with the latter being more motivated to exercise because of a better look (Vašíčková et al., 2014). One study disclosed gender differences in motivation for PA, but their direction depended on motives: health and fitness motives were more prevalent among female students, social status – among male students, and popularity and inaction with friends through sports were similarly important motives for both genders (Ahmed et al., 2020). In general, a systematic review indicated that male adolescents have a higher level of intrinsic motivation towards PA than girls (Cachón-Zagalaz et al., 2023). It has been already well-established that male adolescents are more physically active than females, and although in both genders PA decreases with age, the gap between genders remains almost the same (Sluijs et al., 2021).

Collectively, our results confirmed the assumption that motivation for PA is associated with social support and mental health and in turn translates the effect of the latter to actual PA behavior, regardless of sociodemographic factors and BMI.

*Limitations*: For school students aged 18–19 years, the PA recommendation for 5–17 years old was applied in this study. This was however done intentionally for consistency’s sake considering the entire population of school students as a target group and providing that interventions based on scientific results also won‘t differentiate students <18 and ≥ 18 years. Also, we are not aware of any clear biological rationale or scientific evidence on why the dose of PA for health benefits should change so substantially at passing the calendar age of 18 years by decreasing from 7 h per week for those <18 years to only 4 h 10 min per week for those ≥18 years. Finally, people up to 20 years old are still considered adolescents by some of the classifiers (Sluijs et al., 2021).

## Conclusion

By using a nationally representative sample of Lithuanian school students of both genders, the study identified that moderate-to-vigorous physical activity level of 11–19-year-olds is directly and indirectly related to greater psychological well-being, lower psychological distress, and family but not friends’ support for physical activity.

## Data Availability

Data are available from the corresponding author upon reasonable request.
